# Moderating effect of health-related behavior on the relationship between socioeconomic characteristics and willingness to pay for safe and nutritious food among smallholder farmers in Southwest, Nigeria

**DOI:** 10.3389/fnut.2026.1751765

**Published:** 2026-05-07

**Authors:** Edamisan Stephen Ikuemonisan, Temitope Abiodun Oluwalade

**Affiliations:** Department of Agricultural Economics, Adekunle Ajasin University, Akungba Akoko, Nigeria

**Keywords:** dual system theory, food safety interventions, health-related behavior (HRB), smallholder farmers, socioeconomic factors, willingness to pay (WTP)

## Abstract

**Introduction:**

Smallholder farmers in sub-Saharan Africa face persistent challenges in accessing safe and nutritious food, yet the interplay between socioeconomic constraints and health-related behaviours (HRB) in shaping willingness to pay (WTP) remains underexplored. This study examines how socioeconomic characteristics and HRB jointly influence smallholder farmers’ WTP for safe and nutritious food in Southwest Nigeria. Grounded in Dual System Theory, the research integrates behavioural dispositions with economic decision-making models.

**Methods:**

A cross-sectional survey design was employed, involving 294 respondents across Osun, Ondo, and Ekiti states. Data were collected using structured questionnaires incorporating an adapted Health-Promoting Lifestyle Profile (HPLP) and a Gabor-Granger-based WTP elicitation method. Analytical techniques included Kruskal-Wallis tests and ordinal logistic regression with interaction terms.

**Results:**

Income emerged as the most significant predictor of WTP, with lower-income respondents showing negligible odds of paying premiums for food safety (Wald χ^2^ = 87.969, *p* < 0.001). While HRB alone did not significantly predict WTP (OR = 1.589, *p* = 0.210), it significantly moderated the effects of age (OR = 2.228, *p* < 0.001) and education (OR = 0.251, *p* = 0.023), indicating that HRB can amplify or attenuate the influence of demographic characteristics. Larger household size was positively associated with WTP (OR = 1.245, *p* = 0.009).

**Discussion:**

These results imply that while financial capacity remains central to food-related decisions, behavioural dispositions shape willingness to engage with food safety at a deeper level. This study extends Becker’s household production model by positioning HRB as both an independent and moderating factor in food safety demand. Findings support behaviour-sensitive strategies including: (1) targeted subsidies for households demonstrating strong HRB; (2) mobile-based food safety alert systems; and (3) experiential tools such as vendor-rating applications to enhance food safety access among nutritionally vulnerable smallholder populations.

## Highlights

1. Targeted subsidies: Prioritize households earning below =N221,197 to overcome the affordability barrier.
2. Behavioral nudges for older farmers: Leverage HRB through community health campaigns to boost WTP in aging populations.
3. Family-centric interventions: Design group subsidies or nutrition education for larger households, where collective health drives demand.
4. Rebuild trust with educated consumers: Address cognitive biases (e.g., certification skepticism) via transparent safety labeling and vendor accountability.
5. Dual-pathway policies: Combine economic support (e.g., microloans) with behavioral tools (e.g., habit-forming incentives) for synergistic effects.

## 1 Introduction

The imperative of agricultural intensification, driven by relentless population growth, has placed unprecedented strain on food systems across low- and middle-income countries. While this intensification has bolstered productivity, it has simultaneously amplified concerns regarding the safety of food derived from these systems, particularly fresh produce, dairy, meat, and eggs (1, 2). Persistent inconsistencies in safety protocols throughout agri-food value chains have been directly implicated in the escalating global burden of foodborne diseases (3). Even as global agricultural trade reached a staggering US$2.3 trillion in 2024 (World Trade Organization), the economic toll of foodborne illnesses remains severe, with annual losses estimated at US$50.8 billion in upper-middle-income economies alone (4). The human cost is even more staggering: nearly 600 million people fall ill and over 420,000 die each year from consuming unsafe food, transforming what is often perceived as a supply-chain issue into a profound public health crisis (4).

Nowhere is this crisis more acute than in Nigeria, where the confluence of a vast population, fragmented food systems, and endemic contamination creates a perfect storm of risk. Nearly 23 million Nigerians are affected annually by food safety challenges, a silent epidemic compounded by widespread aflatoxin contamination that disproportionately devastates smallholder farming communities (5). Against this backdrop of persistent health risks, the limitations of traditional economic models in explaining food-related decisions become starkly evident. While classical food economics has long positioned income as the primary driver of dietary choices (6), contemporary evidence from Nigeria reveals a more intricate reality. Financial capacity alone does not sufficiently explain willingness to pay (WTP) for safe and nutritious food (7, 8). Instead, it is the dynamic interplay between income and health-related engagement that unlocks the true potential of purchasing power, reframing food investment decisions not merely as economic transactions, but as behavioral outcomes situated within a broader cognitive-economic interface (9, 10).

This complexity is most pronounced among Nigeria’s smallholder farmers, who operate at the precarious intersection of subsistence living and escalating food safety threats. These households constitute the backbone of the nation’s food system, yet they face a profound paradox: despite spending over 60% of their income on food (11, 12), they continue to suffer from persistent micronutrient deficiencies and widespread aflatoxicosis (13). This apparent contradiction signals a deeper systemic failure, one that conventional economic models fail to capture, namely, the cognitive and behavioral costs of poverty. Under conditions of chronic stress and decision fatigue, which are hallmarks of life below the poverty line, even the most rational health-related behaviors (HRB) often lose their motivational power (14).

The implications of this research, therefore, extend far beyond academic discourse, striking at the heart of Nigeria’s National Food Safety Policy (15) and the implementation of AFCTA’s food trade protocols. The persistent failure of market incentives to curb aflatoxin exposure, despite observed income growth, reveals a fundamental policy blind spot. This study demonstrates that health-related behavior operates as a conditional agent, one that unlocks income’s potential to drive food safety investments. This breakthrough demands innovative policy approaches, including targeted behavioral nudges such as mobile nutrition alerts designed to activate HRB among higher-income farmers (8), and precision-designed, income-tiered vouchers that strategically leverage economic inflection points to amplify HRB’s impact (16). By reconceptualizing HRB as income’s behavioral co-pilot rather than a standalone factor, this study pioneers a new paradigm in food safety economics, one that recognizes the dynamic synergy between economic capacity and health cognition as the true driver of dietary transformation.

The current literature reveals three fundamental blind spots that constrain our understanding of food choice dynamics among vulnerable agrarian populations. First, while health-related behaviors, encompassing health consciousness, nutrition literacy, and food safety practices, are recognized as cognitive mediators of dietary decisions (8), their interaction with income gradients remains a theoretical black box in smallholder agricultural contexts (9, 17). This gap is particularly problematic given emerging evidence of behavioral hysteresis in nutritional economics, whereby past health shocks permanently alter consumption patterns (18). Second, the literature suffers from a critical urban bias (7), leaving rural smallholders, who navigate distinct economic and social realities, woefully understudied. These communities face compounding constraints: erratic income flows that undermine consistent nutrition investment (19), informal markets lacking quality verification mechanisms (20, 21), and deep-seated culinary traditions that create dangerous blind spots in our understanding of their food safety decisions. Third, the literature fails to adequately theorize how HRB might dynamically interact with income to shape WTP. Current frameworks overlook three potentially transformative mechanisms: HRB’s capacity to unlock dormant purchasing power among farmers who have income but lack health motivation (behavioral activation); its ability to enable nutrition-conscious workarounds when income is limited (cognitive substitution); and its role in creating tipping points where income effects suddenly manifest after HRB crosses critical thresholds (phase transition).

To address these gaps, this study moves beyond additive models to examine the synergistic interface between socioeconomic characteristics and health-related behavior in shaping willingness to pay premiums for safe and nutritious foods among Nigerian smallholder farmers. Specifically, the study is designed to achieve three objectives: first, to quantify the relative contributions of socioeconomic factors (age, income, education) and behavioral capital (HRB) in predicting WTP; second, to test whether HRB amplifies or constrains the effects of these socioeconomic characteristics; and third, to identify intervention tipping points where combined economic-behavioral strategies yield super-additive effects on food safety investment. In doing so, the research challenges two pervasive myths in food economics, the simplistic notion that income automatically translates to healthier food choices, and the assumption that behavioral interventions can succeed in economic isolation.

The justification for focusing on smallholder farmers in Southwest Nigeria is both pragmatic and theoretical. This region, while agriculturally rich, faces acute vulnerabilities to food insecurity and malnutrition driven by low income, climate shocks, and limited access to safe, nutritious food (22, 23). Farmers here often experience a disconnect between what they know (rational understanding of health risks) and what they can afford or intuitively choose, making them ideal subjects for a Dual System Theory-based analysis of WTP (24). By focusing on this underserved and strategically important group, the study not only fills a critical research gap but also provides context-specific evidence to guide targeted interventions, such as subsidies and education, that align with national food safety goals and global development priorities, including SDGs 2 (Zero Hunger) and 3 (Good Health and Wellbeing) (25, 26).

## 2 Materials and methods

### 2.1 Research design

This study employed a quantitative, cross-sectional design within a positivist paradigm to examine the synergistic effects of socioeconomic characteristics and health-related behavior on willingness to pay for safe and nutritious food among Smallholder farmers. Guided by deductive reasoning (27), a causal-comparative approach was adopted to analyze relationships between key variables while controlling for confounding factors. The design facilitated the collection of snapshot data, allowing for systematic comparisons across different farming sub-sectors (crop, livestock, and fisheries) in Southwest Nigeria. The Dual System Theory provided the theoretical foundation, ensuring that behavioral and economic influences were adequately captured.

### 2.2 Study area

The study was conducted across three randomly states in Southwest Nigeria, Osun, Ondo, and Ekiti, selected for their socioeconomic and agricultural diversity. These variations ensured a representative sample of Smallholder farmers, defined as smallholders with limited land access (<1 hectare), low mechanization, and unstable incomes (28, 29). The selection criteria emphasized farmers lacking land titles, relying on traditional farming methods, and facing constraints in accessing inputs and markets.

### 2.3 Population, sampling, and bias mitigation

The target population comprised Smallholder farmers engaged in crop, livestock, and fish production. Using Cochran’s (30) formula (Z-score: 1.96, SD: 0.5, margin of error: 0.05), a minimum sample of 384 respondents was determined. A multi-stage sampling technique was applied: first, the 10 Agricultural Development Project (ADP) zones (four ADPs in Ondo State; three in Osun State; and thre in Ekiti state) were selected, followed by random selection of 10 Local Government Areas (LGAs) (one LGA per ADP zone) and 10 villages (one village per LGA). From each village, 39 farmers were sampled across different farming enterprises, totaling 390 respondents (including a six-respondent buffer). To mitigate selection bias, stratified sampling ensured proportional representation of farming sub-sectors, while occupational homogeneity was controlled by excluding non-farming households. Additionally, randomization at the village level minimized geographic bias, enhancing the study’s validity.

The study initially determined a required sample size of 384 using Cochran’s formula but collected 390 responses to account for non-responses, achieving a 77% response rate (*n* = 294). *Post hoc* power analysis confirmed ≥89% power to detect small-to-medium effects, ensuring robust multivariate regression analysis despite the slightly reduced sample. Stratified and randomized sampling enhanced representativeness, while checks for non-response bias and multicollinearity further strengthened validity.

### 2.4 Instrumentation and data collection

The field survey was conducted between 8 March 2025 and 25 March 2025. We distributed a total of 390 questionnaires, achieving an impressive response rate of 77% of the sample size (*n* = 294).

Our structured questionnaire included five validated sections: (i). Demographic and socioeconomic details like age, income, household size and education. (ii). A lifestyle scale tailored for Health-Related Behavior (HRB): The Health-Promoting Lifestyle Profile (HPLP), developed by Walker et al. (31), is a validated scale measuring health-related behaviors across diverse populations, including low-income groups. It is frequently adapted for health/nutrition studies, though context-specific modifications may better suit Smallholder farmers. Hence, it was adapted for this study. A four-point Likert scale (1 = Never, 2 = Sometimes, 3 = Often, 4 = Always) was used to elicit respondent’s perception of their HRB. (iii). Willingness to pay (WTP) based on Gabor-Granger’s work from 1966: Farmers’ WTP for safe food was measured using four price tiers (0%, ≤10%, ≤20%, >20%) via surveys. This tiered approach captured price sensitivity, revealing adoption thresholds and trade-offs between safety and affordability. Responses were analyzed as percentages to identify trends and behavioral links.

### 2.5 Analytical technique

Both Descriptive and Inferential Statistics were employed to analyze and describe the data obtained from this study. One way ANOVA Test was used to explore if the means of income, age, household size and years spent in school across WTP groupings were significant or not. The choice of this tool was because the data were normally distributed. The ordinal regression model is employed to assess the degree to which socioeconomic variables (Income, Age, Education, Household Size) and Health-Related Behavior (HRB) influence the willingness to pay (WTP) for safe and nutritious food. Since the dependent variable (WTP) is ordinal, with naturally ordered response categories (e.g., Not willing, <10% premium, <20% premium, >20% premium), this method is best suited to capture the direction and strength of influence of each independent variable on progressively higher categories of WTP.

### 2.6 Model specification

Let Y denote the ordinal outcome variable (WTP) with k ordered categories. The proportional odds model (also known as the cumulative logit model) is specified as Equation 1:


$$L\,o\,g\,\left(\frac{P\left(Y\leq j\right)}{P\left(Y>j\right)}\right)=\theta_{j}+\beta_{1}\,H\,R\,B+\beta_{2}\,I\,n\,c\,o\,m\,e+\beta_{3}\,A\,g\,e+$$ (1)

$$\beta_{4}\,E\,d\,u\,L\,e\,v\,e\,l+\beta_{5}\,H\,H\,S\,i\,z\,e\,\ldots \,\ldots$$

Where:

i. j = 1,2,3 (since there are 4 WTP categories).

ii. θj is the threshold (cutpoint) for category j.

iii. β_1_to β_5_are regression coefficients for each independent variable.

iv. Income was binned using three cut points of 25%, 50%, and 75%, and Education Level (Pry Education, Secondary Education, Tertiary Education, and Postgraduate Studies).

v. The WTP, a categorical variable captured as follows:

a. Not willing to pay premiums for safe food (0%).

b. Willing to pay up to 10% premium for safe food (<10%).

c. To pay up to 20% premium for safe food (<20%).

d. To pay more than 20% premium for safe food (>20%).

vi. The odds ratio exp(β) indicates the change in odds of being in a higher WTP category for a one-unit increase in the predictor.

To see the interaction effects of the interaction between HRB and Socioeconomic Characteristics on WTP, the model is specified as Equation 2:


$$L\,o\,g\,\left(\frac{P\left(Y\leq j\right)}{P\left(Y>j\right)}\right)=\theta_{j}+\beta_{1}\,H\,R\,B+\beta_{2}\,I\,n\,c\,o\,m\,e+\beta_{3}\,A\,g\,e+$$ (2)

$$\beta_{4}\,E\,d\,u\,L\,e\,v\,e\,l+\beta_{5}\,H\,H\,S\,i\,z\,e+\beta_{6}\,Z\,I\,n\,c\,o\,m\,e^{*}\,Z\,H\,R\,B+$$

$$\beta_{7}\,Z\,A\,g\,e*Z\,H\,R\,B+\beta_{8}\,Z\,E\,d\,u\,L\,e\,v\,e\,l*Z\,H\,R\,B\,\ldots \,\ldots$$

Z stands for standardized score for the selected variables. If the interaction term is not statistically significant, it suggests that the moderating variable (e.g., HRB) does not significantly alter the relationship between the main predictor (e.g., Income) and the outcome (WTP)

### 2.7 Ethical consideration

Even though this is a social behavior inclined study, the authors confirm that the ethical policies of the journal, as noted on the journal’s author guidelines page, have been adhered to. The questionnaire and its administration were approved by the Research and Ethics Committee of Adekunle Ajasin University, Akungba Akoko.

## 3 Results

### 3.1 Description of variables of interest

The descriptive statistics of variables of interest are presented on Table 1. The analysis reveals key characteristics of Smallholder farmers unwilling to pay additional costs for safe and nutritious food. The cohort demonstrates a mean annual income of =N78,068.72 (± = N14,456.11 SD), with education levels averaging 13 years of schooling (±3.4 SD). The typical farmer in this group is 36 years old (±10.5 SD), maintains a household size of seven members (±1.5 SD), and shows moderate health-related behavior (HRB) scores averaging 1.53 (±0.37 SD) on the measurement scale. Notable distribution patterns emerge from the data: income shows perfect symmetry (skewness = 0.00) with a platykurtic distribution (kurtosis = −1.46), while education years exhibit slight positive skewness (0.3). The modal value indicates clustering around =N83,214.02 income, 18 education years, and age 39. The HRB scores range narrowly from 1.00 to 2.09 (range = 1.09), suggesting relatively homogenous health behavior patterns among this subgroup. These findings characterize a population segment facing significant economic constraints (minimum income =N59,772.08) while maintaining basic education and moderate household sizes, factors that likely contribute to their resistance to food safety premiums.

**TABLE 1:** Descriptive statistics.

Not willing to pay premiums for safe food (0%)						Willing to pay up to 10% premium for safe food (<10%)				
	Income	Yrs in sch	Age	HH size	HRB	Income	Yrs in sch	Age	HH size	HRB
Mean	78068.72	13.0	36.0	7.1	1.53	155504.23	12.61	34.00	7.76	1.83
Median	83214.02	13.0	38.0	7.0	1.50	156166.59	12.00	32.00	8.00	1.77
Standard deviation	14456.11	3.4	10.5	1.5	0.37	29711.97	4.55	9.87	1.79	0.48
Kurtosis	−1.46	−1.4	−0.9	−0.4	−0.70	2.33	−0.31	−0.29	0.16	0.67
Skewness	0.00	0.3	0.2	0.2	0.16	−1.12	−0.26	0.56	−0.09	−0.06
Minimum	59772.08	9.0	22.0	5.0	1.00	44869.33	3.00	19.00	2.00	0.33
Maximum	98108.77	18.0	52.0	10.0	2.09	199923.98	22.00	60.00	13.00	2.86
Count	15	15	15	15	15	153	153	153	153	153
**Willing to pay up to 20% premium for safe food (<20%)**						**Willing to pay more than 20% premium for safe food (>20%)**				
	**Income**	**Yrs in sch**	**Age**	**HH size**	**HRB**	**Income**	**Yrs in sch**	**Age**	**HH size**	**HRB**
Mean	201910.11	12.30	37.52	8.35	2.41	258208.07	11.21	37.16	8.26	2.54
Median	208001.90	12.00	36.00	9.00	2.50	259818.34	12.00	37.00	8.00	2.77
Standard deviation	41685.93	4.69	9.86	1.85	0.40	51469.98	6.01	10.55	1.92	0.54
Kurtosis	5.74	−0.48	−0.73	0.45	3.35	7.88	−1.03	−0.67	0.44	1.16
Skewness	−2.58	−0.06	0.56	−0.40	−1.59	−2.46	−0.25	0.30	0.52	−1.41
Minimum	66612.83	3.00	23.00	3.00	1.09	69783.86	1.00	19.00	4.00	1.11
Maximum	235183.39	22.00	56.00	12.00	3.00	329774.39	20.00	56.00	14.00	3.00
Count	69.00	69.00	69.00	69.00	69.00	57.00	57.00	57.00	57.00	57.00

Income, monthly household income (=N); Yrs in sch, years of formal schooling; Age, Age of respondent (years); HH size, household size (number of members); HRB, health-related behavior index (scored 0–3).

The cohort, farmers willing to pay up to 10% premium for safe food (*n* = 153), demonstrates substantially greater economic capacity (mean income = =N155,504.23 ± =N29,711.97) compared to non-paying counterparts, though with notable dispersion (range: =N44,869.33– = N199,923.98). Educational attainment averages 12.61 years (±4.55 SD), while household sizes are moderately larger (mean = 7.76±1.79) than the non-paying group. The population skews younger (mean age = 34.00 ± 9.87) with leptokurtic income distribution (kurtosis = 2.33) and negative skewness (−1.12), indicating clustering toward higher earnings. Health-related behaviors (mean = 1.83 ± 0.48) show improved but still modest engagement, with scores ranging from 0.33 to 2.86. The model values reveal concentration around =N170,250.22 income, 12 education years, age 31, and eight household members. The positive age skewness (0.56) suggests a right-tailed distribution with younger participants, while education’s slight negative skew (−0.26) indicates a minor leftward bias. These parameters characterize a transitional group exhibiting greater but still limited capacity and willingness to invest in food safety.

The data reveal a significantly more affluent demographic profile among farmers willing to pay up to 20% premium, with a mean annual income of =N201,910.11 (± = N41,685.93 SD). This group maintains comparable educational attainment (mean = 12.30 years ± 4.69 SD) but shows distinct characteristics: larger household sizes (mean = 8.35 ± 1.85), older age profile (mean = 37.52 years ± 9.86 SD), and substantially better health-related behaviors (mean HRB = 2.41 ± 0.40). The income distribution exhibits strong negative skewness (−2.58) and high kurtosis (5.74), indicating a concentration of values at the upper range with extreme outliers. Modal values cluster around =N205,683.52 income, 11 education years, age 36, and nine household members. Health behavior scores show negative skewness (−1.59) with leptokurtic distribution (3.35), suggesting most respondents cluster toward the higher end of healthy practices (range: 1.09–3.00). These findings characterize a more economically secure, health-conscious cohort that demonstrates greater willingness to invest in food safety, likely reflecting both greater financial capacity and stronger health awareness.

This table shows the cohort willing to pay the highest premiums (>20%) as the most economically advantaged subgrouped, characterized by a mean annual income of =N258,208.07 ( ± =N51,469.98 SD) that substantially exceeds other WTP categories. While displaying slightly lower mean educational attainment (11.21 years ± 6.01 SD), the group presents distinctive features including a mature age profile (mean = 37.16 years ± 10.55 SD), large household sizes (mean = 8.26 ± 1.92), and the highest health-related behavior scores (mean = 2.54 ± 0.54). The income distribution shows extreme skewness (−2.46) and high kurtosis (7.88), revealing concentration at upper income levels with notable outliers, while education demonstrates a platykurtic pattern (−1.03) with minimal skew (−0.25), indicating relatively uniform educational backgrounds Health behaviors exhibit strong negative skewness (−1.41) and leptokurtic distribution (1.16), with clustering toward optimal practices (mode = 3.00). The near-equality of median (=N259,818.34) and mean incomes, along with consistent household characteristics (mode = 8 members), delineates a unique socioeconomic stratum that combines financial capacity with heightened health consciousness. These findings suggest that while economic resources may be necessary for premium WTP, they prove insufficient alone, as evidenced by this group’s exceptional HRB performance compared to other income-similar cohorts, highlighting the synergistic role of both financial means and health awareness in driving investment in food safety among Smallholder famers.

### 3.2 Comparative analysis of WTP groups

#### 3.2.1 Test of means of socioeconomic characteristics variables across WTP groupings

The ANOVA results presented in Table 2 provide statistical evidence of significant variations in key socioeconomic and health-related characteristics across different willingness to pay (WTP) groups among farmers. Income demonstrated a highly significant difference across WTP groups with F(3, 290) = 150.351, *p* < 0.001, indicating that higher income levels are strongly associated with greater willingness to invest in safe and nutritious food. Similarly, health-related behavior (HRB) exhibited a strong effect, with F(3, 290) = 51.328, *p* < 0.001, suggesting that farmers who engage in healthier practices are considerably more inclined to pay for food safety. Household size also showed a statistically significant variation across WTP groups, F(3, 290) = 3.205, *p* = 0.024, implying that family composition may play a role potentially due to increased concern for household wellbeing or financial strain in larger households.

**TABLE 2:** Output of analysis of variance of socioeconomic variables across willingness to pay (WTP) groupings.

Variable	Sum of squares	df	Mean square	F	Sig.
Income					
Between groups	627787907800.976	3	209262635933.659	150.351	0.000
Within groups	403629106925.023	290	1391824506.638	–	–
Total	1031417014725.999	293	–	–	–
Years spent in school					
Between groups	89.447	3	29.816	1.265	0.287
Within groups	6834.553	290	23.567	–	–
Total	6924.000	293	–	–	–
Age					
Between groups	786.214	3	262.071	2.603	0.052
Within groups	29196.796	290	100.679	–	–
Total	29983.010	293	–	–	–
Household size					
Between groups	31.692	3	10.564	3.205	0.024
Within groups	955.968	290	3.296	–	–
Total	987.660	293	–	–	–
HRB					
Between groups	33.860	3	11.287	51.328	0.000
Within groups	63.769	290	0.220	–	–
Total	97.629	293	–	–	–

Source: Data analysis (2025).

In contrast, years spent in school did not significantly differ across WTP groups, with F(3, 290) = 1.265, *p* = 0.287, suggesting that formal education alone may not sufficiently influence willingness to invest in food safety. Age yielded a borderline result with F(3, 290) = 2.603, *p* = 0.052, slightly exceeding the conventional threshold of significance (*p* < 0.05), indicating a weak or context-dependent relationship. These findings support the theoretical proposition of the Dual-System Theory, wherein both cognitive-rational factors (e.g., income) and intuitive-behavioral processes (e.g., HRB) jointly influence decision-making. The non-significant role of education (years spent in school) challenges the assumption that increased awareness necessarily translates into action. Therefore, policy interventions aiming to improve WTP should prioritize enhancing financial capacity and promoting health-oriented behaviors, especially among low-income farmers, while considering the nuanced roles of household size and age in shaping food safety decisions.

#### 3.2.2 *Post-hoc* tests

Further parametric tests (*post-hoc* tests) were carried out to explore multiple comparisons in socioeconomic characteristics across the WTP premiums for safe food (see Supplementary Appendix VII for detailed post-hoc comparison results), and the results are contained in Supplementary Appendix I.

##### 3.2.2.1 Income

There were statistically significant differences in mean income across all pairwise comparisons of WTP categories (*p* < 0.001 for all). Respondents not willing to pay a premium had significantly lower incomes compared to all other WTP categories. The mean income increased progressively with higher WTP levels. For instance, the difference between WTP group 0 and group 3 was approximately =N180,139, with a 95% CI ranging from =N159,681 to =N200,598. These results provide strong evidence that income is a significant determinant of willingness to pay a premium for safe food.

##### 3.2.2.2 Education (years in school)

Tukey HSD *post hoc* results indicated no statistically significant differences in education levels across WTP categories (*p* > 0.05 in all pairwise comparisons). Despite minor increases in years of schooling with higher WTP, these differences were not meaningful. This suggests that educational attainment may not play a critical role in influencing WTP for safe food, or that the variation in education levels across respondents was insufficient to reveal statistical significance.

##### 3.2.2.3 Health risk behavior (HRB)

Significant differences were found between most WTP groups for HRB scores. Specifically, respondents in group 0 (not willing to pay) had significantly lower HRB scores than those in groups 2 and 3 (*p* < 0.001), and group 1 also differed significantly from groups 2 and 3 (*p* < 0.001). The largest gap was observed between group 0 and group 3, with a mean difference of −1.01, suggesting that higher willingness to pay is associated with greater health-conscious behavior or risk perception. Only the difference between groups 2 and 3 was not statistically significant (*p* = 0.413), indicating that HRB plateaus at higher WTP levels.

##### 3.2.2.4 Household size and age

For household size, no pairwise differences were statistically significant at the 0.05 level, though the contrast between groups 0 and 2 approached significance (*p* = 0.055). This implies that household size may have a marginal influence on willingness to pay, but the evidence is weak. Similarly, age differences across WTP groups were not significant in any pairwise comparison (*p* > 0.05). This indicates that age is not a strong differentiating factor in consumer decisions to pay a premium for safe food in this context.

The analysis shows that both income and health risk behavior (HRB) significantly influence consumers’ willingness to pay (WTP) a premium for safe food. While income reflects economic ability, HRB captures individual health awareness and risk perception. Notably, higher WTP aligns with higher HRB, suggesting that health-conscious individuals are more likely to prioritize food safety. However, income alone does not fully account for WTP differences, as HRB may moderate this relationship. For instance, high-income individuals with low HRB may show low WTP, while low-income individuals with high HRB may still value food safety. This underscores the need for moderation analysis to explore how HRB shapes the income-WTP link, emphasizing the role of behavioral factors in effective food safety policies.

### 3.3 Effects of socioeconomic characteristics on WTP premiums for safe food

Table 3 presents the ordinal regression estimates of socioeconomic predictors on willingness-to-pay premiums. Besides, in the pre-diagnostic tests (Supplementary Appendix II–IV), the model’s deviance value indicated acceptable fit, although the high Pearson Chi-Square ratio suggested potential overdispersion or model misfit, which may require further diagnostic checks. Model fit statistics such as the log-likelihood and AIC provided a reference for accessing the model’s adequacy and comparing alternative specifications. The omnibus likelihood ratio test (Supplementary Appendix III) confirmed that the full model was significantly better than the thresholds-only model, suggesting that the included predictors meaningfully contribute to explaining variations in WTP, with additional robustness and sensitivity analyses reported in Supplementary Appendix VI.

**TABLE 3:** Ordinal regression estimates of socioeconomic predictors on willingness-to-pay premiums.

				95% Wald confidence interval		Hypothesis test				95% Wald confidence interval for Exp (*B*)	
Parameter		*B*	Std. error	Lower	Upper	Wald chi-square	df	Sig.	Exp (*B*)	Lower	Upper
Threshold	(WTP = 0.0%)	−6.185	1.3623	−8.855	−3.515	20.617	1	0.000	0.002	0.000	0.030
	(WTP < 10%)	−0.859	1.3783	−3.561	1.842	0.389	1	0.533	0.424	0.028	6.311
	(WTP < 20%)	3.155	1.3028	0.601	5.708	5.863	1	0.015	23.442	1.824	301.239
HRB		0.833	0.3427	0.161	1.504	5.908	1	0.015	2.300	1.175	4.502
Income											
≤144681.97		−7.542	0.8300	−9.169	−5.915	82.574	1	0.000	0.001	0.000	0.003
144681.98–181503.45		−6.973	0.7941	−8.529	−5.416	77.102	1	0.000	0.001	0.000	0.004
181503.46–221196.76		−4.406	0.6614	−5.702	−3.109	44.373	1	0.000	0.012	0.003	0.045
>221196.77		0^a^	–	–	–	–	–	–	1	–	–
Age		0.020	0.0143	−0.008	0.048	1.882	1	0.170	1.020	0.992	1.049
Education											
Primary education		−0.055	0.6184	−1.267	1.158	0.008	1	0.930	0.947	0.282	3.182
Secondary education		−0.572	0.5195	−1.590	0.446	1.213	1	0.271	0.564	0.204	1.562
Tertiary education: university, polytechnic, NCE		−0.476	0.5236	−1.502	0.550	0.826	1	0.363	0.621	0.223	1.734
Professional and post graduate studies		0^a^	–	–	–	–	–	–	1	–	–
Household size		0.216	0.0786	0.062	0.371	7.585	1	0.006	1.242	1.064	1.448
Scale		1^b^	–	–	–	–	–	–	–	–	–

Source: Data analysis (2025).

^a^Reference category is the baseline (compare all other rows in this group against this one);

^b^This value is not estimated by the data (scale parameter set to 1).

The ordinal regression results in Table 3 offer strong evidence that both economic and behavioral factors significantly influence consumers’ willingness to pay (WTP) for safe food across four ascending payment categories. The model’s threshold parameters reveal statistically significant distinctions between most WTP tiers, particularly between non-payers and premium payers. For instance, the threshold separating the lowest WTP group (WTP = 0%) from higher tiers is highly significant (B = −6.185, *p* < 0.001), as is the upper threshold (WTP = 2.00; B = 3.155, *p* = 0.015). However, the threshold between the lower WTP categories (WTP = 1.00) is not statistically (*p* = 0.533), suggesting weaker discrimination among respondents willing to pay up to 10%.

Income emerged as the most powerful predictor of WTP, with all income categories significantly associated with WTP outcomes (*p* < 0.001 for all binned groups). Compared to the highest income group (bin 4, reference category), individuals in the lowest income bracket (bin 1) were dramatically less likely to pay a premium, with odds reduced by 99.9% (OR = 0.001, 95% CI: 0.000–0.003). Similarly, those in income bins 2 and 3 also had substantially lower odds of higher WTP (OR = 0.001 and OR = 0.012, respectively), demonstrating a strong, monotonic relationship between financial capability and food safety valuation.

Health-related behavior (HRB) was another significant predictor (B = 0.833, *p* = 0.015), indicating that health-conscious individuals are more willing to pay for safe food. Each unit increase in HRB score more than doubled the odds of falling into a higher WTP category (OR = 2.300, 95% CI: 1.175–4.502), reinforcing the critical role of individual health perceptions and behavioral risk aversion in shaping consumer decisions.

Household size also had a modest yet statistically significant effect on WTP (B = 0.216, *p* = 0.006), with each additional household member associated with a 24.2% increase in the odds of paying more for food safety (OR = 1.242, 95% CI: 1.064–1.448). This may reflect greater collective concern for family health or shared food consumption risks in larger households.

In contrast, age (*p* = 0.170) and education (all *p* > 0.271 across bins) did not significantly predict WTP. Age had only a minimal positive association (OR = 1.020), while education levels failed to show meaningful variation in WTP, suggesting that formal schooling does not translate directly into heightened food safety valuation within this population.

Overall, the findings underscore that economic capacity and health-conscious behavior are the primary drivers of willingness to pay for food safety, while demographic characteristics like age and education have limited predictive utility. These results emphasize the need for policies that not only enhance consumers’ economic means but also cultivate health awareness, particularly among low-income individuals, to strengthen engagement with food safety initiatives.

### 3.4 Moderating effects of HRB in the relationship between socioeconomic characteristics and WTP

Table 4 presents ordinal regression estimates of the moderating effect of HRB on the relationship between socioeconomic and willingness-to-pay premiums, with full model diagnostics and extended interaction estimates provided in Supplementary Appendix V. The ordinal regression model shows excellent fit (Likelihood Ratio χ^2^ = 358.194, *p* < 0.001; Deviance/df = 0.442) with robust information criteria (AIC = 355.981, BIC = 425.969). Income emerges as the strongest predictor (Wald χ^2^ = 87.969, *p* < 0.001), followed by household size (χ^2^ = 6.874, *p* = 0.009), while HRB’s main effect is insignificant (χ^2^ = 1.568, *p* = 0.210). Significant moderation occurs for age-HRB interaction (χ^2^ = 17.440, *p* < 0.001), showing HRB’s amplifying effect strengthens with age, while income-HRB effects remain independent (χ^2^ = 6.214, *p* = 0.102). Education-HRB interaction shows marginal significance (χ^2^ = 8.141, *p* = 0.043), indicating context-dependent effects. The findings reveal economic factors predominantly drive WTP, with HRB’s moderating role varying across socioeconomic dimensions, being strongest for age.

**TABLE 4:** Ordinal regression estimates of the moderating effect of HRB on the relationship between socioeconomic and willingness-to-pay premiums.

				95% Wald confidence interval		Hypothesis test				95% Wald confidence interval for Exp (*B*)	
Parameter		*B*	Std. error	Lower	Upper	Wald chi-square	df	Sig.	Exp (*B*)	Lower	Upper
Threshold	(WTP = 0.0%)	−8.376	1.6228	−11.557	−5.196	26.644	1	0.000	0.000	9.568E-6	0.006
	(WTP < 10%)	−2.629	1.5182	−5.604	0.347	2.998	1	0.083	0.072	0.004	1.415
	(WTP < 20%)	1.974	1.4769	−0.921	4.868	1.786	1	0.181	7.196	0.398	130.094
Education											
Primary education -PRY EDU		−0.176	0.6585	−1.467	1.115	0.072	1	0.789	0.839	0.231	3.048
Secondary education – SEC EDU		−0.121	0.6554	−1.406	1.163	0.034	1	0.853	0.886	0.245	3.201
Tertiary education: university, polytechnic, NCE – TERT-EDU		−0.267	0.5897	−1.423	0.889	0.205	1	0.651	0.766	0.241	2.432
Professional and post graduate studies – POST GRAD		0^a^	–	–	–	–	–	–	1	–	–
[PRY EDU*HRB (binned) = 1]		0.101	0.4597	–0.800	1.002	0.048	1	0.827	1.106	0.449	2.723
[SEC EDU*HRB (binned) = 2]		–1.091	0.5113	–2.093	–0.089	4.556	1	0.033	0.336	0.123	0.915
[TERT-EDU^HRB (binned) = 3]		–1.380	0.6059	–2.568	–0.193	5.191	1	0.023	0.251	0.077	0.825
[POST GRAD*HRB (binned) = 4]		0^a^	–	–	–	–	–	–	1	–	–
Income											
≤144681.97		−8.558	0.9333	−10.387	−6.729	84.092	1	0.000	0.000	3.082E-5	0.001
144681.98–181503.45		−7.647	0.8879	−9.387	−5.907	74.183	1	0.000	0.000	8.378E-5	0.003
181503.46–221196.76		−4.726	0.7190	−6.136	−3.317	43.210	1	0.000	0.009	0.002	0.036
>221,196.77		0^a^	–	–	–	–	–	–	1	–	–
[≤144681.97*HRB (binned) = 1]		−0.912	0.5611	−2.011	0.188	2.639	1	0.104	0.402	0.134	1.207
[144,681.98 – 181,503.45*HRB (binned) = 2]		0.106	0.5939	−1.058	1.270	0.032	1	0.859	1.111	0.347	3.560
[181503.46 - 221196.76*HRB (binned) = 3]		−0.241	0.5210	−1.262	0.781	0.213	1	0.644	0.786	0.283	2.183
[>221,196.77*HRB (binned) = 4]		0^a^	–	–	–	–	–	–	1	–	–
HRB		0.463	0.3696	−0.262	1.187	1.568	1	0.210	1.589	0.770	3.278
Age		0.026	0.0161	−0.006	0.057	2.565	1	.109	1.026	0.994	1.059
AGE*HRB		0.801	0.1918	0.425	1.177	17.440	1	0.000	2.228	1.530	3.244
Household size		0.219	0.0835	0.055	0.383	6.874	1	0.009	1.245	1.057	1.466
Scale		1^b^	–	–	–	–	–	–	–	–	–

Source: Data analysis (2025).

^a^Reference category is the baseline (compare all other rows in this group against this one);

^b^This value is not estimated by the data (scale parameter set to 1). “*” means interaction signs.

The ordinal regression analysis provides statistically robust evidence about the determinants of willingness-to-pay (WTP) premiums among Smallholder farmers. The threshold parameters reveal a clear differentiation between non-payers and payers, with the strongest demarcation for absolute non-payers (B = −8.376, *P* < 0.001). This large negative coefficient indicates a substantial baseline resistance to paying premiums, which must be overcome by other factors. The non-significant thresholds for intermediate and high premium categories suggest this payment levels are less psychologically distinct for respondents.

Income emerges as the most powerful determinant of WTP, showing a strong negative relationship across all income levels. The extremely small odds ratios (OR = 0.000 for the lowest income bin) indicate that lower income farmers have near-zero odds of being in higher WTP categories compared to the reference group. The progressively larger OR values across income bins (0.000 → 0.009) demonstrates a gradient effect where each income increment substantially reduces, but does not eliminate, the negative impact on WTP. This suggests that while all farmers in the study population are income-constrained, the degree of constraint varies meaningfully across income levels.

Household size shows a significant positive relationship with WTP (B = 0.219, *p* = 0.009). The odds ratio of 1.245 indicates that each additional household member increases the odds of being in a higher WTP category by 24.5%. This may reflect either greater food needs in larger households or more pooled financial resources enabling premium payments. The effect size suggests this is a meaningful but secondary influence compared to income.

The age-HRB interaction reveals important behavioral economics insights (B = 0.801, *p* < 0.001). The OR of 2.228 means that for each year of age, the effect of HRB on WTP more than doubles. This suggests that health-conscious behaviors become increasingly important drivers of food safety investments as farmers age, possibly due to greater health awareness or accumulated savings. The large effect size indicates this interaction may be practically significant for targeting interventions.

Education-HRB interactions show counterintuitive negative effects. For secondary educated farmers, health behaviors actually reduce WTP (B = −1.091, OR = 0.336), meaning their odds of paying premiums decrease by 66.4% when HRB is considered. The effect is even stronger for tertiary educated respondents (B = −1.380, OR = 0.251). This suggests education may create skepticism about premium food value or redirect limited resources to other priorities. These significant negative interactions (*p* = 0.033 and 0.023, respectively) require careful consideration in intervention design.

The non-significant HRB main effect (*p* = 0.210) and income-HRB interactions (all *p* > 0.05) provide important null findings. These indicate that health behaviors alone don’t drive WTP, and that income constraints operate independently of health consciousness. This has crucial policy implications, suggesting that behavioral interventions may need to be paired with economic support to be effective.

## 4 Discussion

This study investigates how socioeconomic characteristics and health-related behavior (HRB) interact to influence willingness to pay (WTP) for safe and nutritious food among Smallholder farmers in Southwest Nigeria. Using a cross-sectional, quantitative design grounded in Dual System Theory, the research analyzes 294 responses from smallholder farmers across Osun, Ondo, and Ekiti states. Key objectives include quantifying the individual and interactive effects of income, education, age, and HRB on WTP, and identifying intervention thresholds where combined strategies yield super-additive effects. Data were collected using structured questionnaires covering demographics, HRB (via an adapted Health-Promoting Lifestyle Profile), and tiered WTP levels based on Gabor-Granger pricing. Analytical methods included ANOVA tests and ordinal regression, with interaction terms to explore how HRB moderates socioeconomic influences on WTP. The study provides empirical insights for designing integrated policies, such as subsidies coupled with behavioral nudges that enhance food safety adoption among nutritionally vulnerable, low-income farming populations.

### 4.1 Income as a foundational driver of WTP

The cumulative logit model identified income as the most powerful and consistent determinant of willingness to pay (WTP) for safe and nutritious food. With a Wald χ^2^ of 87.969 (*p* < 0.001), income exerted a steep and inverse effect on the odds of paying premiums, particularly among lower-income respondents. Those earning ≤ = N221,196.76 had odds ratios ranging from 0.000 to 0.009, indicating a near-zero likelihood of WTP in the lowest brackets. This finding affirms Becker’s (6) theory of household production, which posits that economic resources fundamentally shape the ability to invest in health capital. Notably, the declining odds across progressively higher income bins reflect the non-linear threshold dynamics of health-related expenditure, revealing an economic tipping point beyond which demand for food safety becomes viable.

### 4.2 Health-related behavior: no main effect but crucial moderation

Although health-related behavior (HRB) did not exhibit a significant main effect on WTP (OR = 1.589, *p* = 0.210), its conditional influence emerged through interaction effects, supporting Mullainathan and Shafir’s (10) scarcity theory. Their framework suggests that under resource constraints, behavioral drivers such as HRB may remain dormant until financial or cognitive bandwidth allows for their activation. The non-significant main effect indicates that HRB, by itself, does not uniformly influence food safety decisions; however, it becomes instrumental in specific demographic and cognitive contexts, as revealed through moderation analysis.

### 4.3 Household size and the role of collective health decision-making

The variable household size showed a significant positive effect on WTP (OR = 1.245, *p* = 0.009), implying that larger families are more likely to prioritize food safety, potentially due to a collective concern for group health. This partially aligns with Lustig (32) insights into poverty dynamics, though it nuances their view by suggesting that communal health priorities may sometimes supersede budgetary constraints. In the Nigerian context, where extended family units are common, this finding highlights the importance of designing family-centered food safety interventions, such as household-level nutrition education or community-focused subsidies.

### 4.4 The cognitive limits of age and education

Surprisingly, age (*p* = 0.109) and education, particularly secondary education (*p* = 0.853), were not significant predictors of WTP. This diverges from findings by Akinwehinmi et al. (17), Alimi (33), who identified education as a central determinant of food safety behaviour. The inconsistency may be explained by Reyna’s (34) assertion that in informal markets, intuitive judgements (System 1 thinking) often override analytical reasoning (System 2), especially when vendor reputation and trust substitute for formal certifications. This reinforces the argument that cognitive heuristics dominate decision-making in low trust, high-risk food environments like Nigeria’s.

### 4.5 HRB’s moderating role on age and education

The moderation analysis underscored HRB’s context-dependent power. The interaction between HRB and age (MODAGEHRB) was highly significant (OR = 2.228, *p* < 0.001), indicating that older individuals with strong health behaviors were over twice as likely to pay premiums. This supports Epstein’s (35) cognitive-experiential theory, which posits that personal experience amplifies health-conscious behavior in later life. Conversely, the HRB × tertiary education interaction had a significantly negative effect (OR = 0.251, *p* = 0.023), suggesting that educated individuals with strong HRB may paradoxically be less likely to pay. This finding may be explained by cognitive dissonance (36) and the Dunning-Kruger effect, where educated individuals overestimate their ability to detect unsafe food, thus distrusting formal certifications, a pattern echoed in Wu et al. (21), Odera (20).

### 4.6 Parallel effects of HRB and income

While the HRB × Income interaction was not statistically significant (*p* = 0.102), its marginal value indicates a parallel, not moderating, role. This is consistent with Kahneman’s (24) Dual System Theory, which posits that intuitive and deliberative processes often function independently. Here, HRB and income each contribute to WTP, but do so along separate cognitive pathways. This finding challenges conventional models that assume linear moderation and instead supports a dual-driver model of food safety decisions.

### 4.7 Policy implications and theoretical contributions

This study makes significant contributions to both theoretical understanding and policy design by extending Becker’s (6) income-deterministic model of household behavior. While income remains a foundational driver of willingness to pay (WTP) for safe and nutritious food, our findings demonstrate that health-related behavior (HRB) exerts a conditional influence rather than acting as a direct predictor. Specifically, HRB amplifies the effect of age (OR = 2.228), suggesting that older individuals with strong health-conscious behaviors are more likely to invest in food safety. In contrast, HRB suppresses the influence of education (OR = 0.251), implying that more educated individuals with high HRB may paradoxically distrust formal safety labels, especially in environments prone to certification fraud. Finally, the near-significant independence of HRB from income dynamics (*p* = 0.102) suggests that behavioral drivers operate alongside, rather than subordinate to, economic capacity.

### 4.8 Counterintuitive findings and behavioral insights

Several counterintuitive findings merit further reflection. The negative HRB-education interaction (OR = 0.251) suggests that educated consumers may over-rely on personal judgement, echoing the Dunning-Kruger effect. Additionally, middle-aged farmers (36–47 years), rather than the elderly, were more inclined to pay premiums, supporting Sopi et al. (37), who argue that the demographic balances health awareness with earning capacity. Finally, the consistent significance of household size (OR = 1.245) suggests it is a more robust predictor of WTP than education, positioning it as a key targeting variable for group-based interventions.

## 5 Conclusion

This study advances behavioral food economics by quantifying HRB’s direct and moderating roles in WTP, revealing critical income thresholds for policy leverage. It challenges neoliberal assumptions that income alone drives food safety investment, instead showing HRB as income’s “behavioural co-pilot,” a paradigm shift for Sub-Saharan Africa’s nutrition policies. Furthermore, health-related behavior (HRB) acts as a hidden lever, amplifying WTP among older farmers while paradoxically reducing it among the educated, likely due to distrust in formal certifications. Household size also emerges as a key predictor, suggesting communal health concerns can outweigh financial constraints. These findings challenge traditional economic models by showing that behavioral and cognitive factors interact with, but do not replace, income as the foundational determinant of food safety investment.

## Data Availability

The original contributions presented in this study are included in the article/Supplementary materials, further inquiries can be directed to the corresponding author.
