# Supplementary material for: Moderating effect of health-related behavior on the relationship between socioeconomic characteristics and willingness to pay for safe and nutritious food among smallholder farmers in Southwest, Nigeria
Source: Front Nutr. 2026 May 7;13:1751765. doi: 10.3389/fnut.2026.1751765 (PMC13189762; doi:10.3389/fnut.2026.1751765)
Supplement: Supplementary file 1 [file Supplementary_file_1.pdf]

Appendix I

Multiple Comparisons

Dependent Variable: Income

Games-Howell

		Mean Difference			95% Confidence Interval	
(I) WTP	(J) WTP	(I-J)	Std. Error	Sig.	Lower Bound	Upper Bound
.00	1.00	-77435.51282	4438.68059	.000	-89566.3861	-65304.6395
	2.00	-123841.39748	6254.29802	.000	-140326.0934	-107356.7015
	3.00	-180139.35347	7772.28466	.000	-200597.6461	-159681.0608
1.00	.00	77435.51282	4438.68059	.000	65304.6395	89566.3861
	2.00	-46405.88465	5563.65376	.000	-60941.4720	-31870.2973
	3.00	-102703.84065	7228.16770	.000	-121724.7768	-83682.9045
2.00	.00	123841.39748	6254.29802	.000	107356.7015	140326.0934
	1.00	46405.88465	5563.65376	.000	31870.2973	60941.4720
	3.00	-56297.95600	8465.26825	.000	-78390.5006	-34205.4114
3.00	.00	180139.35347	7772.28466	.000	159681.0608	200597.6461
	1.00	102703.84065	7228.16770	.000	83682.9045	121724.7768
	2.00	56297.95600	8465.26825	.000	34205.4114	78390.5006

Dependent Variable: Education

Tukey HSD

		Mean Difference			95% Confidence Interval	
(I) WTP	(J) WTP	(I-J)	Std. Error	Sig.	Lower Bound	Upper Bound
.00	1.00	.39216	1.31347	.991	-3.0018	3.7861
	2.00	.69565	1.38301	.958	-2.8780	4.2693
	3.00	1.78947	1.40877	.583	-1.8508	5.4297
1.00	.00	-.39216	1.31347	.991	-3.7861	3.0018
	2.00	.30350	.70398	.973	-1.5156	2.1226
	3.00	1.39732	.75333	.250	-.5493	3.3439
2.00	.00	-.69565	1.38301	.958	-4.2693	2.8780
	1.00	-.30350	.70398	.973	-2.1226	1.5156
	3.00	1.09382	.86892	.590	-1.1515	3.3391
3.00	.00	-1.78947	1.40877	.583	-5.4297	1.8508
	1.00	-1.39732	.75333	.250	-3.3439	.5493
	2.00	-1.09382	.86892	.590	-3.3391	1.1515

Dependent Variable: HRB

Tukey HSD

		Mean Difference			95% Confidence Interval	
(I) WTP	(J) WTP	(I-J)	Std. Error	Sig.	Lower Bound	Upper Bound
.00	1.00	-.30037	.12687	.086	-.6282	.0275
	2.00	-.88270	.13359	.000	-1.2279	-.5375
	3.00	-1.01224	.13608	.000	-1.3639	-.6606
1.00	.00	.30037	.12687	.086	-.0275	.6282
	2.00	-.58232	.06800	.000	-.7580	-.4066

	3.00	-.71187	.07277	.000	-.8999	-.5238
2.00	.00	.88270	.13359	.000	.5375	1.2279
	1.00	.58232	.06800	.000	.4066	.7580
	3.00	-.12954	.08393	.413	-.3464	.0873
3.00	.00	1.01224	.13608	.000	.6606	1.3639
	1.00	.71187	.07277	.000	.5238	.8999
	2.00	.12954	.08393	.413	-.0873	.3464

. The mean difference is significant at the 0.05 level.

Multiple Comparisons

Dependent Variable: HRB

Tukey HSD

		Mean Difference			95% Confidence Interval	
(I) WTP	(J) WTP	(I-J)	Std. Error	Sig.	Lower Bound	Upper Bound
.00	1.00	-.30037	.12687	.086	-.6282	.0275
	2.00	-.88270	.13359	.000	-1.2279	-.5375
	3.00	-1.01224	.13608	.000	-1.3639	-.6606
1.00	.00	.30037	.12687	.086	-.0275	.6282
	2.00	-.58232	.06800	.000	-.7580	-.4066
	3.00	-.71187	.07277	.000	-.8999	-.5238
2.00	.00	.88270	.13359	.000	.5375	1.2279
	1.00	.58232	.06800	.000	.4066	.7580
	3.00	-.12954	.08393	.413	-.3464	.0873
3.00	.00	1.01224	.13608	.000	.6606	1.3639
	1.00	.71187	.07277	.000	.5238	.8999
	2.00	.12954	.08393	.413	-.0873	.3464

. The mean difference is significant at the 0.05 level.

Multiple Comparisons

Dependent Variable: Household Size

Games-Howell

		Mean Difference			95% Confidence Interval	
(I) WTP	(J) WTP	(I-J)	Std. Error	Sig.	Lower Bound	Upper Bound
.00	1.00	-.63137	.41472	.445	-1.8028	.5401
	2.00	-1.21449	.44775	.055	-2.4493	.0203
	3.00	-1.12982	.46473	.095	-2.4006	.1409
1.00	.00	.63137	.41472	.445	-.5401	1.8028
	2.00	-.58312	.26505	.129	-1.2731	.1069
	3.00	-.49845	.29282	.328	-1.2643	.2674
2.00	.00	1.21449	.44775	.055	-.0203	2.4493
	1.00	.58312	.26505	.129	-.1069	1.2731
	3.00	.08467	.33799	.994	-.7962	.9655
3.00	.00	1.12982	.46473	.095	-.1409	2.4006
	1.00	.49845	.29282	.328	-.2674	1.2643
	2.00	-.08467	.33799	.994	-.9655	.7962

Dependent Variable: Age
Games-Howell

(I) WTP	(J) WTP	Mean Difference (I-J)	Std. Error	Sig.	95% Confidence Interval Lower Bound	Upper Bound
.00	1.00	2.00000	2.82482	.892	-6.0538	10.0538
	2.00	-1.52174	2.95836	.955	-9.8116	6.7681
	3.00	-1.15789	3.04899	.981	-9.6229	7.3071
1.00	.00	-2.00000	2.82482	.892	-10.0538	6.0538
	2.00	-3.52174	1.43031	.071	-7.2437	.2002
	3.00	-3.15789	1.60942	.210	-7.3669	1.0511
2.00	.00	1.52174	2.95836	.955	-6.7681	9.8116
	1.00	3.52174	1.43031	.071	-.2002	7.2437
	3.00	.36384	1.83372	.997	-4.4160	5.1437
3.00	.00	1.15789	3.04899	.981	-7.3071	9.6229
	1.00	3.15789	1.60942	.210	-1.0511	7.3669
	2.00	-.36384	1.83372	.997	-5.1437	4.4160

Appendix II

Goodness of Fit[a]

	Value	df	Value/df
Deviance	355.076	726	.489
Scaled Deviance	355.076	726	
Pearson Chi-Square	11787.862	726	16.237
Scaled Pearson Chi-Square	11787.862	726	
Log Likelihood[b]	-177.538		
Akaike's Information Criterion (AIC)	379.076		
Finite Sample Corrected AIC (AICC)	380.186		
Bayesian Information Criterion (BIC)	423.278		
Consistent AIC (CAIC)	435.278		

Dependent Variable: WTP
Model: (Threshold), HRB, Income (Binned), Age, Education (Binned), Household Size
a. Information criteria are in smaller-is-better form.
b. The full log likelihood function is displayed and used in computing information criteria.

Appendix III

Omnibus Test[a]

Likelihood Ratio Chi-Square	Df	Sig.
321.099	9	.000

Dependent Variable: WTP
Model: (Threshold), HRB, Income (Binned), Age, Education (Binned), Household Size

a. Compares the fitted model against the thresholds-only model.

Appendix IV

Tests of Model Effects

Source	Type III Wald Chi-Square	df	Sig.
HRB	5.908	1	.015
Income (Binned)	89.447	3	.000
Age	1.882	1	.170
Education (Binned)	2.111	3	.550
Household Size	7.585	1	.006

Dependent Variable: WTP

Model: (Threshold), HRB, Income (Binned), Age, Education (Binned), Household Size

Appendix V

Goodness of Fit[a]

	Value	Df	Value/df
Deviance	317.981	719	.442
Scaled Deviance	317.981	719	
Pearson Chi-Square	3829.851	719	5.327
Scaled Pearson Chi-Square	3829.851	719	
Log Likelihood[b]	-158.990		
Akaike's Information Criterion (AIC)	355.981		
Finite Sample Corrected AIC (AICC)	358.755		
Bayesian Information Criterion (BIC)	425.969		
Consistent AIC (CAIC)	444.969		

Dependent Variable: WTP

Model: (Threshold), Education (Binned), MODEDUHRB (Binned), Income (Binned), MODINCHRB (Binned), HRB, Age, MODAGEHRB, Household Size

a. Information criteria are in smaller-is-better form.

b. The full log likelihood function is displayed and used in computing information criteria.

Appendix VI

Omnibus Test[a]

Likelihood Ratio Chi-Square	df	Sig.
358.194	16	.000

Dependent Variable: WTP

Model: (Threshold), Education (Binned), MODEDUHRB (Binned), Income (Binned), MODINCHRB (Binned), HRB, Age, MODAGEHRB, Household Size

a. Compares the fitted model against the thresholds-only model.

Appendix VII

Tests of Model Effects

Source	Type III Wald Chi-Square	df	Sig.
Education (Binned)	.295	3	.961
MODEDUHRB (Binned)	8.141	3	.043
Income (Binned)	87.969	3	.000
MODINCHRB (Binned)	6.214	3	.102
HRB	1.568	1	.210
Age	2.565	1	.109
MODAGEHRB	17.440	1	.000
Household Size	6.874	1	.009

Dependent Variable: WTP

Model: (Threshold), Education (Binned), MODEDUHRB (Binned), Income (Binned), MODINCHRB (Binned), HRB, Age, MODAGEHRB, Household Size
